# Exploring how occupational therapists and physiotherapists evaluate rehabilitation potential of older people in acute care

**DOI:** 10.1177/03080226211011386

**Published:** 2021-05-05

**Authors:** Gemma Bradley, Katherine Baker, Catherine Bailey

**Affiliations:** Faculty of Health and Life Sciences, 5995 Northumbria University, Newcastle Upon Tyne, UK

**Keywords:** Rehabilitation potential, professional reasoning, older people, qualitative research, ethnography

## Abstract

**Introduction:**

Evaluations of rehabilitation potential are an everyday occurrence, yet the concept is poorly understood and there is a lack of understanding about the reasoning process. This study aimed to explore how occupational therapists and physiotherapists evaluated the rehabilitation potential of older people following an acute hospital admission.

**Method:**

Focused ethnography was utilised, primarily using observation, interviewing and review of records within one acute medical ward in a general hospital in the United Kingdom. Five patient participants gave consent for their episode of care to be studied, for interactions with professionals to be observed and for their clinical records to be reviewed. Three occupational therapists and two physiotherapists then participated in individual interviews.

**Findings:**

Thematic analysis of data led to the identification of a four-stage reasoning process. The four stages are as follows: gathering baseline information; provision of curative and supportive interventions; provision and monitoring of rehabilitative interventions; the evaluation of rehabilitation potential and decision about the subsequent pathway.

**Conclusions:**

The reasoning process illustrates the professional reasoning of occupational therapists and physiotherapists when evaluating rehabilitation potential for older adults in acute care. However, it also highlights vulnerabilities to professional reasoning which may contribute to subjectivity, inconsistency or risk to patients.

## Introduction

A rehabilitative phase of care in hospital plays a pivotal role in helping older people to recover after an acute admission (Chang &Wang, 2013), and in the aftermath of COVID-19, rehabilitation is becoming the longer-term priority to assist people to recover from lengthy hospital admissions and significant functional decline (Royal College of Occupational Therapists, 2020). Not only will rehabilitation be the focus for individuals recovering from the COVID-19 illness and associated deconditioning, but the wider population are likely to have broader rehabilitation needs linked to the disruption of normal health and care services (DeBiase et al., 2020).

The evaluation of *rehabilitation potential* is said to be an everyday occurrence in hospitals and amongst rehabilitation professionals (Cunningham, Horgan & O’Neill, 2000). Such evaluations help to determine when and if rehabilitation begins, the intensity of rehabilitation required and at what point rehabilitation may fail to deliver meaningful outcomes (Burton et al., 2015). The post-COVID-19 need for rehabilitation has been described as a likely tidal wave (DeBiase et al., 2020), and determining if rehabilitation is required and who is most likely to benefit, within already pressurised systems, has never been more important.

Evaluations of rehabilitation potential are extremely significant for patients, families and professionals and have been linked to the allocation of rehabilitation resources (Zhu et al., 2007; Burton et al., 2015; Arling et al., 2000). There are examples of the presence of rehabilitation potential being cited as part of service admission criteria (Kotiadis et al., 2004) and people being excluded from services on the basis of no rehabilitation potential (National Audit Office, 2010).

Burton et al. (2015) also highlight wider reasons as to why evaluations of rehabilitation potential are of significance, recognising the emotional impact for health professionals and an awareness that judgements about potential can become self-prophesying. Yet despite the significance, Enderby et al. (2017) highlight limited understanding of professional reasoning and an absence of recognised tools or algorithms to support decision-making. Enderby et al. (2017) go on to compare evaluations of rehabilitation potential to a ‘guessing game’ (p709).

### Literature review

Rehabilitation potential has been defined as an estimate of the individual’s capability of cooperating with a rehabilitation programme and making measured functional gains (Rentz, 1991). Zhu et al. (2007) define rehabilitation potential retrospectively if a person has made functional improvement or remained at home over a period of 1 year and discuss that an effective assessment of rehabilitation potential relates to the selection of individuals who are most likely to benefit from rehabilitation. Burton et al. (2015) suggest that professionals describe the concept of rehabilitation potential by referring to two main elements—the visible achievement of goals or outcomes over time and the observation of carry-over within and across therapy.

Definitions in other studies are perhaps notable by their absence or ambiguity. Shun et al. (2017) attempt to explore factors influencing perceptions of rehabilitation potential but do not clearly define how they are interpreting this foundation concept. Other studies allude to judgements about rehabilitation potential with limited explanation of what this means or entails (Kotiadis et al., 2004; Kumlien et al., 1999).

There are a small number of studies which aim to explore professional reasoning in relation to evaluating rehabilitation potential and, within this small number, most relate to specific clinical pathways for conditions such as stroke and traumatic brain injury. Burton et al. (2015) used multi-professional focus groups to discuss a hypothetical case scenario relating to rehabilitation potential following stroke. Findings highlighted that judgements about potential tend to emerge from observing responsiveness to therapy, even through potential failure or poor outcomes, rather than from predictor variables prior to starting rehabilitation.

Shun et al. (2017) recruited 12 occupational therapists to identify the most important patient-related factors when considering rehabilitation potential in people following acquired brain injury. The group agreed 11 factors as essential to consider when evaluating rehabilitation potential: age, behaviour, cognitive abilities, endurance, home environment, medical status, observed improvement in acute care post-injury, physical abilities, post-injury functional status, pre-injury functional status and patient and family expectations. However, alongside these patient-related factors, other factors were noted including the organisational context (such as time and resource pressures), professional expertise, experiential knowledge, knowledge of scientific evidence and ethical considerations. There are also studies which compare evaluations of rehabilitation potential between different professional groups (Cunningham, Horgan and O’Neill, 2000) and between staff and care home residents (Chang et al., 2011) with both studies suggesting poor agreement between groups and a lack of shared understandings.

With the stakes being high for patients, families and professionals, the lack of clarity about the concept of rehabilitation potential and the limited understanding of the reasoning process emerged as important and underdeveloped areas within the existing literature. More specifically, understanding such issues in relation to older people with complex and heterogeneous rehabilitation needs was an additional important focus. The aim of this study was to understand the reasoning process of occupational therapists and physiotherapists when evaluating rehabilitation potential of older people following acute hospital admissions.

## Method

To meet the study aim, focussed ethnography was utilised, guided by principles of social constructionism. Social constructionism supports the notion that meanings are developed within social contexts, and individuals and groups construct meanings and ways of understanding through their social interactions and shared language (Burr, 2003). Moreover, from this constructionist position, people are thought to act towards external realities based on such meanings (Blumer, 1969). The ways in which individuals and groups act and behave are socially constructed and framed by a set of physical, temporal, social and political circumstances (Burr, 2003). Dewey (1929; p136) suggests that the test of ideas and of thinking is found in the consequences of the acts to which the idea leads which supports the position that understanding evaluations of rehabilitation potential can be effectively understood by studying the actions and decisions in context.

From this social constructionist perspective, ethnography enables the study of cultural groups in their natural setting to understand the realities of actions within social contexts and in real time (Creswell, 2009). More specifically, focussed ethnography is appropriate for focussing on specific groups and distinct issues within cultures (Roper and Shapira, 2000). These principles were utilised in this study to focus on one particular ward environment and explore the practices of occupational therapists and physiotherapists when evaluating rehabilitation potential of identified patients.

The study was approved by the Faculty of Health and Life Sciences Research Ethics Committee at Northumbria University (HLS-PHW141515) and the NHS Research Ethics Committee (15/NE/0322).

Fieldwork was based within one medical ward within a general hospital in the United Kingdom. The 28-bedded ward had a remit to provide care for adult patients who were admitted for medical reasons and did not follow other established pathways within the hospital system, such as stroke or orthopaedics. Although not exclusively for older people, due to the demographics of the patient group requiring unplanned hospitalisation, the ward primarily cared for patients over the age of 65 years for a wide range of reasons including falls, delirium and infections. The ward was often referred to by staff as both a medical ward and a care of the elderly ward. The ward team consisted of medical staff, qualified and assistant nursing staff, physiotherapists, occupational therapists and social workers, supported by a wide range of other teams and services such as pharmacy, dietetics and specialist teams for issues such as tissue viability and old-age psychiatry.

There were three phases of fieldwork over a period of 14 months. Collecting data using multiple methods and at different time points is seen as a strength of ethnographic work and an approach which increases credibility through triangulation of methods and theoretical frameworks (Brewer, 2000). Phase one took place in April 2016 and was a 2-week orientation phase. Following this, between May and July 2016, phase two was an 8-week ‘patient-tracking’ phase focussing on the practice of occupational therapists and physiotherapists involved with identified patients. Finally in April–May 2017 (after a break of 10 months to enable preliminary data analysis), phase three was a 4-week period including in-depth interviews with occupational therapists and physiotherapists involved in phase two. Various methods were used to generate data—recognised as a feature of ethnography (Rock, 2001)—primarily observation and interviews, supported by detailed fieldnotes. All observations and interviews were undertaken by one researcher.

During phase one, written consent was gained from all nursing, occupational therapy (OT) and physiotherapy staff working on the identified ward. Public notices were displayed for patients, families and staff who moved between wards to let them know that observations were taking place. Before any observation, the researcher introduced herself, explained the purpose of observations and asked for verbal consent to be present. During phase two, the same processes remained in place although in addition, the researcher gained written consent to observe specific elements of care with identified patients and to access clinical records. One patient was deemed to lack capacity to consent, and therefore, a family member was approached to act as a consultee. Because of the time period between phase two and phase three, occupational therapists and physiotherapists who had been involved in phase two were asked to renew their written consent for the in-depth interviews during phase three.

To identify the patient cases, the researcher utilised principles of purposive sampling (Polgar and Thomas, 2013) to approach patients who met inclusion criteria outlined in Table 1. Screening for patients who met these criteria was undertaken by the researcher through attendance at daily multidisciplinary handover meetings.

**Table 1.** Inclusion criteria for patient participants in phase two observations.

Essential criteria	Additional purposive sampling criteria to reflect characteristics of inpatient population
Aged over 65 years	At least two participants aged over 85 years
Current functional level below preadmission functional level and determined to have rehabilitation needs by the healthcare team	At least one participant with cognitive impairment
Medically fit to be approached to participate as determined by the healthcare team	
Able to communicate in English	

Data were analysed using thematic analysis to report and interpret patterns (Braun and Clarke, 2006). Familiarisation with the data took place over the whole data collection period with shorthand researcher fieldnotes translated in to longhand accounts and with the addition of analytical notes and reflections. These notes were combined with interview transcripts and catalogued both chronologically and in relation to different patient episodes. Following this familiarisation stage, initial codes were developed such as instances involving key language (e.g. ‘rehabilitation’, ‘rehabilitation potential’ and ‘baseline’); instances of attempting to predict outcomes; instances relating to OT and physiotherapy; information exchange between professionals, or with patients and families and instances which highlighted timing and chronology of decisions or actions. The data were reviewed in line with these codes, and themes were identified. Although the analysis was predominantly undertaken by one researcher, data were shared with other analysts who represented different disciplines and methodological positions to increase credibility of analysis (Patton, 2015). After these stages, final themes were named and this subsequently led to the identification of four stages of reasoning.

### Findings

After the initial orientation of phase one, phase two involved tracking five patient cases with multiple periods of observation both of direct health professional, patient and relative interactions and also of meetings and discussions about the identified patients. Three occupational therapists and three physiotherapists were involved in this phase of fieldwork. Of the six professionals involved in phase two, three occupational therapists and two physiotherapists then participated in phase three interviews. One physiotherapist had changed roles within the organisation and did not reply to the request to participate in phase three.

**Figure 1.**
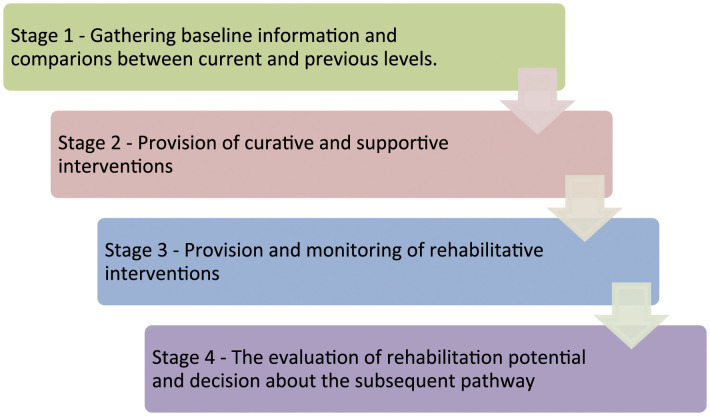
Four-stage reasoning process for evaluating rehabilitation potential.

Data analysis led to the identification of a four-stage reasoning process which is summarised in Figure 1 and described in subsequent sections. In the presentation of findings, where there is certainty about exact words (e.g. from verbatim extracts taken from audio-recorded interviews), double quotation marks are used. Where the researcher relied on her own recall and translations within fieldnotes, single quotation marks are used.

### Gathering baseline information and comparisons between current and previous levels

Understanding of baseline information was discussed by health professionals as the first step in evaluating rehabilitation potential. ‘Baseline’ was a term used across all professionals in the setting and interpreted to relate to a patient’s previous level of health, disability, function and social support. During fieldwork, one physiotherapist summarised ‘it is looking at the baseline, what has got them to that baseline or if that is just their norm for a long time and whether they are likely to get back to that taking in to mind factors such as comorbidities and cognition’.

Baseline information included new and pre-existing health conditions; secondary issues such as pain, pressure or presence of delirium; movement and mobility levels; functional ability and care needs and information about home environment and social support. Through the review of clinical records, this information gathering commenced during the initial admission and medical assessment (often on other wards or sites) and then continued through a series of discipline-specific assessments. Information about particular aspects relating to pre-admission function was included in both physiotherapy and OT assessment documentation (for example, pre-admission mobility and transfers).

Despite the significance of information about baseline function, the responsibility to gather comprehensive baseline information was not always clear in the patient cases observed, and gaps and inconsistencies were noted in clinical records. The use of the ‘?’ symbol was often noted with entries such as ‘? usual level of function’ or ‘? baseline’. For one patient, this was still being used on day 14 of the admission with the entry ‘? has reablement or ? long-term care package’. For another patient, a physiotherapy entry on day one documented that the person was normally mobile with a stick, but on day seven, there was a further entry to indicate the patient normally walked with a tripod. A further example was noticed where within an initial nursing assessment, a comment of ‘no problems’ was noted in relation to continence prior to admission, although on day seven there was an entry which documented regular incontinence.

This stage also involved comparison between current and previous function. The expression ‘getting back to baseline’ was a frequent part of professional discourse and regularly discussed as an overarching goal in handover and multidisciplinary meetings and in many informal interactions between health professionals. During observations, one physiotherapist discussed that the evaluation of rehabilitation potential ‘is a judgement about getting as close to baseline as possible’. An occupational therapist framed this in their own words saying the evaluation is judging ‘what they are achieving and where they were before, and what’s the likelihood of getting any better’. It was also not just occupational therapists and physiotherapists who thought about rehabilitation potential in this way; in a multidisciplinary team meeting, one of the ward doctors also described rehabilitation potential as ‘the potential to get back to baseline’.

On occasion, professionals did progress from gathering baseline information to an evaluation of rehabilitation potential and judgements about an appropriate pathway, bypassing the intervening second and third stages. For example, an occupational therapist shared that one patient had ‘known dementia, an Mini-Mental State Examination (MMSE) of 9 out of 30 and she was not coping prior to admission…[we] would just be taking up a rehab bed’. For this patient, the decision was made quickly to begin the process for a move to 24-h care.

### Provision of curative and supportive interventions

Occupational therapists and physiotherapists discussed the impact that acute medical issues had on reaching judgements about rehabilitation potential and that attempts were therefore made to treat issues which could potentially be resolved or improved. Professionals also inferred that this in turn informed and improved the judgement about rehabilitation potential.

During one discussion, a physiotherapist reflected on a past case, suggesting that ‘if I’d seen him in writing as to when he first got here…I would have thought he is going to take a week or two…but it all depends on…getting rid of the delirium’. And the same physiotherapist, when discussing one of the patients tracked during the research, mentioned ‘…some of the pressure issues she has got on her legs, so that’s going to obviously affect any mobility progression. But that’s now under treatment which is a medical issue’. Time for interventions to resolve medical issues such as pain or constipation, or to support wider needs such as nutrition and sleep were seen as integral in not just assisting with a judgement about whether someone had the potential and capacity to benefit from rehabilitation interventions, but also assisted with judging the time they may need to respond.

### Provision and monitoring of rehabilitative interventions

The third stage involved providing rehabilitative interventions to work towards goals such as sitting out of bed, improving transfers or mobility or working towards improving capacity to manage functional activities such as toileting and dressing.

Although some curative, supportive and rehabilitative interventions were observed as happening simultaneously, a sequential element was implied in that the impact of rehabilitative interventions, and therefore meaningful judgements about rehabilitation potential, could not be evaluated until the team had worked towards potential resolution of acute problems. An example was discussed within a weekly multidisciplinary team meeting where the occupational therapist asked the team if they needed to prepare the family that the patient was unlikely to make functional improvements and therefore a return home was unlikely. At this point, the consultant stated ‘she has been unwell…needs more time. [We will] review in a week and see what kind of progress we are making’.

The importance of engagement in a rehabilitation process in order to make meaningful judgements about the likelihood of making functional gains was observed. In relation to one patient, the physiotherapist discussed ‘it is early days yet and we will see what happens…so until I see that progress, that initial this is where we are…I can then make that decision (about the patient’s rehabilitation potential)’.

In relation to the same patient, during a weekly MDT the consultant discussed that she was ‘not sure what her rehab potential is…give her 2 weeks and review’. She also added that the team would be guided by ‘OT and physio’ during this time. This was reinforced by a written record of the meeting which stated ‘more time for rehab—review 2/52’.

### The evaluation of rehabilitation potential and decision about the subsequent pathway

Although not a linear process, the stabilisation of acute medical issues, and the monitoring of the response to rehabilitative interventions, subsequently informed a more confident evaluation of likely progress and decisions about where a patient would go next or which (if any) services they would be referred to. Pathway decisions on this ward included patients remaining on the ward for a period of rehabilitation; patients transferred to a different rehabilitation ward; patients transferred to a bed-based intermediate care unit; patients transferred to a specialist unit for cognitive assessment and rehabilitation; patients discharged home with referral to another service who could provide rehabilitation (most often a community reablement service) and patients discharged home or to another care environment with no further rehabilitation. From all of the services involved in these pathways, the only service with written criteria to inform judgements about who to refer was the community reablement service suggesting that many pathway decisions relied on tacit and potentially subjective reasoning.

This evaluation of likely progress and the decision about the pathway were difficult to separate and are therefore presented as one stage. During one interview, a participant response suggested blurred lines between the judgement about rehabilitation potential and the judgement about the appropriateness of a transfer:

‘There was a patient who got sent up to one ward and then brought back again because their rehab potential…I would say that the therapists were probably right there but they got over-ruled by the medical team’ (HP3).

The same participant went on to highlight that rehabilitation potential could almost be considered as a criterion for a transfer of care but once again recognised the ambiguity:

‘One of the things I always say to people when they send them to (rehab facility) is “they’ve got to have rehab potential” (laughs)’. (HP3).

One of the critical challenges suggested during fieldwork observations was that the evaluation of rehabilitation potential could potentially be used as a strategy to promote movement within the system:

‘Sometimes I think it was said, well, if it was going to be quite a complex discharge if there was a lot of problems, it seemed they will be like they need more time so we will send them to rehab when there was not really a rehab need’ (HP4).

At this stage, the consideration of access to finite rehabilitation resources—such as availability of beds or waiting lists for specific services—was observed as being influential in the reasoning of practitioners.

## Discussion

Through an examination of real-time actions and discussions of occupational therapists and physiotherapists, four distinct stages of reasoning were identified (as outlined in Figure 1). The literature review highlighted that previous studies have utilised retrospective or hypothetical scenarios and have tended to focus on specific patient groups or pathways such as stroke; therefore, examination of this reasoning process in real time and for a heterogeneous group of older people has enabled important extensions to knowledge.

It is perhaps unsurprising that the reasoning process began with an information gathering stage to compare current with previous function. In simple terms, this information helps to illuminate what change may be possible for a patient—what level of function they could potentially return to post injury, illness or admission or what level of care requirements the person received prior to admission and would be realistic to aim to get back to.

Significantly, this first stage indicates the beginning of a predictive reasoning process, using predictive factors such as presence of comorbidities and pre-admission cognition. Predictive reasoning is one reasoning strategy recognised as pertinent to rehabilitation professionals when required to envision future scenarios based on estimated responses to therapy (McGlinchey and Davenport, 2015). Predictive factors such as presence of cognitive impairment and a higher number of comorbidities are generally accepted to project more limited rehabilitation outcomes (Hershkovitz et al., 2010; Press et al., 2007; Semel et al., 2010), and there is an acknowledged evidence base in support of this reasoning strategy. However, many of these studies are specific to particular patient pathways such as hip fracture (Press et al., 2007; Semel et al., 2010) or stroke (Hakkennes et al., 2013) and are therefore difficult to generalise to an older population with heterogeneous needs. Also, although the presence of some of these factors may lead to poorer rehabilitation outcomes, rehabilitation gains are still reported which suggest that rehabilitation potential may be affected but not eradicated by factors such as cognitive impairment (Elphick et al., 2007).

Information about previous level of cognitive function presented as significant, with examples where this led to a rapid evaluation of limited rehabilitation potential and judgements that further rehabilitation resources would have limited benefit. Yet critics have challenged the sensitivity of a tool such as the MMSE (Ballard, 2006) and in recent guidelines for Intermediate Care and Reablement, it was specifically highlighted that people should not be excluded from services because of pre-existing conditions such as dementia (NICE, 2017). Whilst pre-existing cognitive impairment has been associated with poorer rehabilitation outcomes, the reasons are likely to be deeply layered and multifactorial, with one suggestion being that this group may be less likely to be given access to rehabilitation interventions (Longley et al., 2019). Examples of rapid evaluations of rehabilitation potential which circumnavigated the provision of rehabilitation interventions or monitoring the response suggest there may be some truth in this suggestion.

This first stage highlighted that the expression of aiming to ‘get back to baseline’ was an established part of professional lexicon in this context and was observed to be embedded within the reasoning process of professionals when evaluating rehabilitation potential. Interestingly, guidance about hospital discharge in England during the COVID-19 pandemic challenges professionals working in acute hospitals to avoid expressions ‘such as back to baseline’ (p31; Department of Health 2020) and link this to guidance that only essential medical interventions (rather than those focussing on optimising function) should require bed-based hospital care. Although the study presented in this article was carried out between 2016 and 2017 and professional practice, language and reasoning is likely to have evolved, the extent to which expressions about ‘back to baseline’ were used in practice and observed to be influencing reasoning points to areas which are important areas for reflection.

Furthermore, the emphasis placed on getting back to baseline was suggestive that this became an implicit goal and priority within the reasoning process with the subsequent evaluation of rehabilitation potential becoming an evaluation of the likelihood of achieving functional improvement. Hammell (2006) suggested that physical improvement can become a preoccupation of rehabilitation professionls. The extent to which improvements towards a pre-admission baseline may be prioritised at the expense of wider environment, psychological and social needs raises important questions in practice.

Stages two and three suggest separation between the provision of curative and rehabilitative interventions and that reasoning involves monitoring responses to these separate interventions. Separation of these functions of care has roots in international health policy (OECD, 2011) and can be seen in the many models of care which separate acute and rehabilitation phases (Bachmann et al., 2010). Yet critics highlight a crucial challenge that patients may end up receiving medical or rehabilitation interventions but not both at the same time (Wade, 2016). The quote from one interview participant that people get physically moved within the system if medical needs are felt to outweigh rehabilitation needs may cause unnecessary risks and distress for patients and families. There is also the risk that people wait longer than necessary for rehabilitative interventions if this is seen as something that happens separately or at a later stage.

Evidence in some specialisms suggests that the co-location of acute and rehabilitation services may result in better functional outcomes (Chan et al., 2014), and this may support services to reorganise to reflect this. It would be interesting to explore whether the reasoning processes of professionals working in such environments result in less separation between monitoring responses to curative and rehabilitative interventions. Importantly, if rehabilitation interventions happened simultaneously during curative and supportive phases, then the judgement about rehabilitation potential arguably becomes less significant because it is inevitably less entwined with when phases should begin and end.

The importance of the third stage of providing and monitoring rehabilitation interventions emphasises that, in most instances, professionals do not rely on predictive factors alone to evaluate rehabilitation potential but instead combine this with the information they gather through engagement in a rehabilitative process. This was echoed by Burton et al. (2015) where professionals consistently described the visible achievement of goals over time and the observation of carry-over within and across therapy as important influences on their evaluation of rehabilitation potential. Significantly, Burton et al. (2015) also raise the critical issue that it is therefore the availability, and receipt, of rehabilitation interventions which may influence rehabilitation outcomes adding further complexity to the evaluation of rehabilitation potential. Considering professionals have expressed that they feel they may deliver suboptimal versions of rehabilitation due to systemic constraints in acute care (Bradley et al., 2019), then the final evaluation of potential in stage four is arguably at risk of becoming a forecast of limited potential because of limited rehabilitation opportunities.

The fourth stage involved a final judgement of rehabilitation potential and a judgement about which service or pathway might best enable potential to be optimised. However, participant quotes highlight that the evaluation is far from confident or definitive, reflecting the subjectivity and ambiguity reflected in other studies. This ambiguity, alongside the fact that the judgement about potential and the judgement about the pathway were difficult to separate, is of interest here. The blurring of these elements suggests an inevitability that the decision about rehabilitation potential becomes heavily influenced by availability of beds and services, and evaluations potentially become about manoeuvring people within systems (Dodier and Camus, 1998). The fact that the concept is ambiguous and subjective means this is open to bias and whether someone is referred for a rehabilitation bed or service could vary dramatically between patients, driven by pressures on services or professionals at any given time.

Underpinning the reasoning process in its entirety, are principles of ethical reasoning—a reasoning strategy used to attempt to resolve ethical dilemmas or to balance one or more values against another in an effort to act in the best interests of service users (Chapparo and Ranka, 2008). Competing ethical principles of deontology and utilitarianism is perhaps inevitable, with the former being driven by the intention to do good for all, and the latter aiming to produce the greatest good for the greatest number within finite resources (Hugman, 2005). This push and pull between ethical demands and principles has been recognised in other studies about rehabilitation decision-making (Levack, 2009) and is likely to contribute to ethical dilemmas and tensions for health professionals (Bushby et al., 2015).

Whilst there are examples of generic models and frameworks to describe and explore professional reasoning (Dreyfus and Dreyfus, 1986; Higgs and Jones, 2000), this four-stage process potentially provides a starting point to articulate professional reasoning specifically when evaluating rehabilitation potential. In their expert commentary, Enderby et al. (2017) emphasise the challenges of ambiguity and subjectivity in this area and raise the lack of recognised decision-making tools or algorithms as a critical issue in practice. Although not claiming to be a decision-making tool or sophisticated algorithm, this could provide a framework to aid discussions with patients and families, discussions between professionals and to support documentation of evaluations of rehabilitation potential. It could also be used as a framework to support education of students and reflective practice amongst practitioners, providing a structure around what may often be unstructured ideas, to reflect on decisions and to learn from experience (Bannigan and Moores, 2009).

Previous studies to explore evaluations of rehabilitation potential have tended to rely on analysis of hypothetical or retrospective patient cases, and therefore, the use of immersive and real-time methods to examine this issue is viewed as a particular strength of this research. An additional strength includes the intentional focus given to examining this issue in relation to older people who have heterogeneous needs and non-uniform pathways and where extensions to knowledge are significant for many practitioners.

Limitations of the study include the small sample sizes both in relation to patient cases and health professional participants which lead to questions about transferability outside of the immediate localised setting. However, Hammersley (1998) suggests that ethnographic research should instead be viewed in terms of relevance, with readers encouraged to judge the importance of the topic and the contribution to existing knowledge. An additional limitation is that the focus on OT and physiotherapy meant that wider perspectives of patients, families and multiple professionals were not fully understood. It is perhaps here where another limitation of the reasoning process is evident in that the collaborative context of professional reasoning does not receive emphasis although again, this was reflective of how reasoning was being enacted in practice.

## Conclusion

Findings led to the identification of a four-stage reasoning process, which could have important utility in practice to assist practitioners to make their reasoning explicit to patients, families, other professionals and to themselves, and assist with both written and verbal communication. However, while the process provides an overview of what may be happening from authentic practice examples, the description itself illustrates some of the challenges when evaluating rehabilitation potential, such as separating curative, supportive and rehabilitative interventions, associating the evaluation of rehabilitation potential with a movement within the health or care system or limited involvement of patients and families in decision-making. Examples of language being out of alignment with contemporary guidance is also a challenge but highlights areas where a cultural change may be needed within practice to reframe commonly used terminology and reasoning strategies. Therefore, the reasoning process is not presented as a representation of best practice but instead as an authentic reflection of reasoning in this context and would benefit from refinement in collaboration with practitioners, patients and families. Only then could it become a meaningful tool to aid articulation of decision-making with potential to reduce subjectivity and ambiguity in this important aspect of professional reasoning.

## Key findings

1. A four-stage reasoning process was evident when occupational therapists and physiotherapists evaluated the rehabilitation potential of older people in acute care.
2. This reasoning process helps to identify influences on, and complexities within, evaluations of rehabilitation potential

## What the study has added

This study provides insight into evaluations of rehabilitation potential for older people and has placed importance on understanding this from the perspective of occupational therapists and physiotherapists.
